# *Brachypodium* *sylvaticum*, a Model for Perennial Grasses: Transformation and Inbred Line Development

**DOI:** 10.1371/journal.pone.0075180

**Published:** 2013-09-20

**Authors:** Michael A. Steinwand, Hugh A. Young, Jennifer N. Bragg, Christian M. Tobias, John P. Vogel

**Affiliations:** 1 Western Regional Research Center, United States Department of Agriculture, Agricultural Research Service, Albany, California, United States of America; 2 Department of Plant & Microbial Biology, University of California, Berkeley, California, United States of America; 3 Department of Plant Sciences, University of California Davis, Davis, California, United States of America; USDA-ARS, United States of America

## Abstract

Perennial species offer significant advantages as crops including reduced soil erosion, lower energy inputs after the first year, deeper root systems that access more soil moisture, and decreased fertilizer inputs due to the remobilization of nutrients at the end of the growing season. These advantages are particularly relevant for emerging biomass crops and it is projected that perennial grasses will be among the most important dedicated biomass crops. The advantages offered by perennial crops could also prove favorable for incorporation into annual grain crops like wheat, rice, sorghum and barley, especially under the dryer and more variable climate conditions projected for many grain-producing regions. Thus, it would be useful to have a perennial model system to test biotechnological approaches to crop improvement and for fundamental research. The perennial grass *Brachypodium* *sylvaticum* is a candidate for such a model because it is diploid, has a small genome, is self-fertile, has a modest stature, and short generation time. Its close relationship to the annual model *Brachypodium* *distachyon* will facilitate comparative studies and allow researchers to leverage the resources developed for *B* *. distachyon* . Here we report on the development of two keystone resources that are essential for a model plant: high-efficiency transformation and inbred lines. Using *Agrobacterium tumefaciens*-mediated transformation we achieved an average transformation efficiency of 67%. We also surveyed the genetic diversity of 19 accessions from the National Plant Germplasm System using SSR markers and created 15 inbred lines.

## Introduction

Perennial grasses are widely grown for forage and turf. In addition, they promise to serve as a significant source of renewable energy in the near future [1,2]. In these contexts, a perennial lifecycle offers several obvious advantages over an annual lifecycle [1,3]. First, the rapid speed with which perennial grasses reach canopy cover in the spring means that perennials intercept more solar radiation than annuals. This helps explain the greater biomass accumulation of perennials. Second, perennials require less energy input per year since they are not replanted each year. This greatly improves the net energy balance (the ratio of the energy from the fuel produced to the energy used to make that fuel) of biofuels obtained from perennial grasses. Third, perennials require less fertilizer because they remobilize nutrients into the roots during senescence. This reduces pollution due to nutrient runoff, reduces costs and further improves the net energy balance, as it takes a lot of energy to make fertilizer. Fourth, perennial culture reduces erosion and increases soil organic matter relative to annual crop production since the soil is only bare during the establishment year. Finally, their deeper root systems allow perennials to access water from deep soil horizons that are not accessible to annual crops, decreasing the need for irrigation and increasing the acreage that can be farmed without irrigation. Thus, it is no surprise that perennial grasses are predicted to account for approximately 16 billion gallons of renewable fuels by the year 2022 [1,2].

In contrast to their dominance as forage and biomass crops, perennial grasses are rarely grown as grain crops [4]. Since perennial grasses once dominated many grasslands the question arises as to why they are almost nonexistent as modern grain crops. It is commonly believed that because of the tradeoff between carbon allocation to reproductive structures (the grain) and the vegetative structures necessary for perenniality (roots, rhizomes, etc.), annual grains have a theoretical advantage over perennials in terms of grain yield. However, a careful examination of this hypothesis reveals little supporting evidence [4,5]. While there may be a yield penalty during the establishment period of a perennial crop, only the first year for a grass, yield in later years can be equal to or greater than that for annual crops. Indeed, in their review Van Tassel et al. [5] assembled a table of the reproductive effort of several very high yielding perennial crops and the most productive crop (apples) allocated a remarkable 65% of the dry matter produced each year to reproductive structures [6]. This compares very favorable to the average, 48%, and maximum, 67%, harvest index reported for maize [7]. Thus, there is nothing inherently incompatible between a perennial lifecycle and high yield of reproductive structures. Indeed, a strong argument can be made that by beginning to grow as early in the season as possible and by growing more rapidly due to an established root system perennial grains could have higher yields than their annual counterparts. This naturally leads to the question of why annual grains dominate modern agriculture. In a review of this subject Van Tassel et al. convincingly argue that domestication of annual grains was favored because of the way and conditions in which early humans first domesticated these crops [5]. Briefly, planting seeds from annual grasses for the next harvest would bring about steady genetic changes in the annual grass because plants that produced more seeds or larger seeds would be favored for planting and have a selective advantage. By contrast, genetic enhancement of perennial grasses would be slow because early people would not need to plant every year. If they did plant seeds, the existing perennial root systems would limit the effect of planted seed on beneficial allele frequency. The inter-tangled root systems of many individuals would also mean that it would be impossible to select a superior vegetative clone as is done with other perennial crops like fruit trees.

Since, modern breeding techniques, especially marker assisted selection, do not suffer from the limitations encountered by early humans, it should be possible to breed high-yielding perennial grains that use less water, fertilizer and reduce erosion when compared to annual grains [4,8]. Several groups are already working toward this goal ( [9,10,11,12,13]; www.landinstitute.org). If successfully developed, perennial grains would have many of the same benefits as perennial forage and biomass crops outlined above (e.g. higher yield, less fertilizer, less water, etc.). These advantages may be especially important as humanity struggles to feed a growing population [14], especially since the climates of many major grain-producing regions are predicted to become dryer, hotter and more variable. Under these conditions, a deeper perennial root system would be a major advantage.

Given the importance of perennial grasses as forage, biomass and potentially grain crops, a truly tractable perennial model system (compact, self fertile, easily transformed, rapid initial generation time, etc.) is needed to accelerate the acquisition of knowledge about all aspects of the perennial lifecycle. This information could then be used to design rational breeding strategies. For example, if markers existed for the genes responsible for maintaining meristems and inducing dormancy, they could be selected for during the breeding of perennial grains. Likewise, if markers existed for genes that promote extensive rhizome production, they could be selected against to produce perennial grain crops with fewer tillers and larger grains. Another use of a perennial model grass would be to evaluate biotechnological improvements in a perennial context. This is especially important for traits that may affect the onset of dormancy or other traits that could affect winter survival. For example, modulating cytokinin levels has been shown to increase stress tolerance and yield in annual crops [15,16], but altering cytokinin levels could conceivably have an adverse effect on perenniality. In this context, it is noteworthy that while some perennial grasses (e.g. *Lolium* *perenne* , *Agrostis* *stolonifera* *,* *Panicum* *virgatum* ) can be efficiently transformed [17,18,19,20,21,22,23], their polyploidy, self incompatibility and/or large size greatly decreases their suitability as model systems [2].

*Brachypodium* *sylvaticum* is an excellent candidate for a model perennial grass because it is self-fertile, small, diploid, easy to grow and has a small genome. Evolutionarily the genus *Brachypodium* lies midway between rice and wheat [24,25,26] and *B* *. sylvaticum* has already been used as a genomic model for wheat. Indeed, the first bacterial artificial chromosome library in the genus *Brachypodium* was made from *B* *. sylvaticum* and *B* *. sylvaticum* markers have been used to clone several wheat genes [27,28,29,30]. A *B* *. sylvaticum* model system would benefit from the large collection of resources developed for the closely related annual model grass *Brachypodium* *distachyon* (for a recent review see [31]:). Comparative studies of these closely related annual and perennial species might be particularly useful to identify and test candidate ‘perenniality’ genes. Here we describe a highly efficient transformation system for *B* *. sylvaticum* , the development of inbred lines and genetic characterization of the diversity of accessions in the NPGS collection.

## Materials and Methods

### Plant materials and growth conditions

Seeds from 23 accessions were obtained from the USDA National Plant Germplasm System (Table 1). Plants were grown in a peat-based potting mix (Sunshine mix 1, Sun Gro Horticulture, http://www.sungro.com/) and fertilized with a time release fertilizer (Osmocote Plus 15-9-12, Scotts Co., http://www.scotts.com). After sowing, pots were placed at 4°C for 1 or 2 weeks to promote synchronous germination. Growth conditions were the same as used for *B* *. distachyon* [32]. Briefly, plants were grown in growth chambers with a 20 hr light: 4 hr dark light cycle and in a greenhouse without shading but with daylength extended to 16 hours by supplemental lighting.

**Table 1:** Lines used in this study.

**NPGS accession number**	**Inbred line name**	**map location^1^**	**Collection location from NPGS passport data**	**C-value (pg/nucleus**)	**chromosome number**	**time to flowering^3^ (weeks**)	**maximum inflorescence height (cm**)
**PI172383**	Sin-1	1	Sinop, Turkey	0.91	nd	never	81
**PI204863**	Zig-1	2	Zigana, Turkey	1.07	nd	5	72
**PI204865**	Tra-1	3	Trabzon, Turkey	1.05	nd	8	88
**PI206546**	na	4	Greece	1.61 1.48	27	never	na
**PI206619**	na	5	Samsun, Turkey	1.49	nd	never	na
**PI251102**	Vel-1	6	Veles, Macedonia	1	nd	6	83
**PI268222**	Gor-1	7	Gorgan, Iran	1.02 0.92	nd	10	na
**PI269842**	Ain-1	8	Ain Draham, Tunisia	0.96 0.95	18	9	132
**PI287787**	na	9	Toledo Province, Spain	2.1	36	never	na
**PI297868**	Aus-1	na^2^	Australia^2^	0.98	18	8	107
**PI318962**	Can-1	10	Candeleda, Spain	1.02	nd	8	108
**PI325218**	na	11	Stavropol, Russia	0.85	nd	never	na
**PI344569**	na	12	Slovakia	1.46	27	never	na
**PI345965**	Osl-1	13	Oslo, Norway	0.91	nd	9	86
**PI380758**	Ard-1	14	Ardebil, Iran	0.9	nd	9	127
**PI384810**	Ast-1	15	Astara, Iran	0.93	nd	8	124
**PI440175**	Alm-1	16	Almaty, Khazakstan	0.79	nd	5	91
**PI564896**	Nov-1	17	Novosibirsk, Russia	1.01	nd	5	95
**PI610793**	Vlo-1	18	Vlore, Albania	0.97	nd	never	116
**PI636630**	Kry-1	19	Krym, Ukraine	0.96	nd	8	105
**W6 23501**	na	20	Xizang, China	nd	nd	never	na
**W6 23524**	na	21	Xizang, China	0.95	nd	never	na
**W6 23538**	na	22	Xizang, China	nd	nd	never	na

^1^Map locations are based on the nearest town according to the passport data. If no town was indicated, the location is the center of the country.

^2^This location may be an error and was not included in the map because the passport data indicates that the species collected was *Agropyron* *caninum* and the natural range of *B* *. sylvaticum* does not include Australia. Thus, the original stock may have been switched with another *B* *. sylvaticum* accession.

^3^Plants were stratified/vernalized for 2 weeks after sowing and then grown in a growth chamber under 20 hr daylight conditions. “Never” means the plants did not flower within 14 weeks.

### Inbred line development

Seed heads for single seed descent were bagged (Lawson 217, Lawson Bag Company, http://www.lawsonbags.com/) prior to anthesis to prevent cross-pollination. Lines were inbred for 2-5 generations depending on the generation time. Line names consist of the first three letters of the town near the collection location or the country of origin. Inbred lines were not made for accessions that did not flower under our conditions.

### SSR analysis

For each accession, DNA was extracted from one of the initial plants grown from the seed obtained from NPGS prior to any inbreeding, though not necessarily the individual plant that was used for inbreeding. Lyophilized leaf tissue was ground to a powder using a Retsch MM400 Ball Mill (www.retsch.com) at 30 cycles/sec for 1 min, then mixed with 500 uL of extraction buffer (0.1M Tris-HCL pH 7.5, 0.05 EDTA pH 8.0, 1.25% SDS) and incubated at 65°C for 30 minutes, mixing every 10 minutes by inversion. 250 uL of 6M ammonium acetate was added and samples were mixed and incubated on ice for 15 minutes. After centrifugation at 13,000 rpm for 5 minutes at room temperature, 600 uL of the supernatant was collected. 360 uL of isopropanol was added and DNA precipitated for at least 1 hour on ice. Samples were then centrifuged for 10 minutes at 14,000 rpm at room temperature to pellet DNA. The DNA pellet was rinsed with 70% ethanol, dried and resuspended in water. SSRs were detected and analyzed exactly as described previously [33].

### Tissue culture conditions

**Figure 1:**
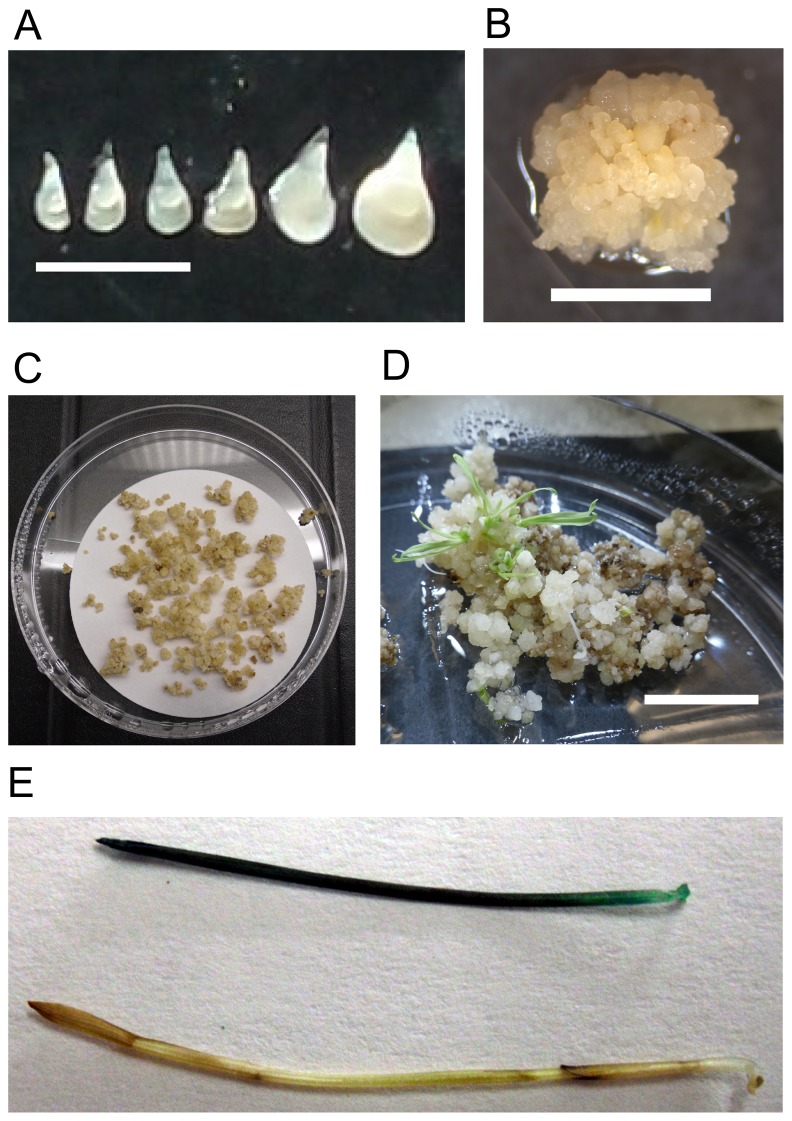
Tissue culture stages of inbred line Ain-1. (a) Embryos dissected from immature seeds. The five embryos on the left will produce high quality embryogenic callus. The embryo on the right is slightly too large. Note that it is also more opaque. The scale bar is 1 mm. (b) Appearance of high-quality embryogenic callus. Note the structures that look like embryos. Scale bar is 5 mm. (c) Callus on dry filter paper after removal from *Agrobacterium* suspension. The plate is 10 cm in diameter. (d) Transgenic plantlets regenerating from callus. Note the presence of darker non-transgenic callus that is dying. Scale bar is 1cm. (e) GUS positive (top) and negative (bottom) plants representative of the transgenic outcrossed progeny and non-transgenic selfed progeny of the non-transgenic plants used to determine outcrossing frequency.

Immature seeds were surface sterilized as described [34]. Immature embryos were dissected from immature seeds under a dissecting microscope in a laminar flow hood and placed scutellum side up (Figure 1a) on callus induction media (per L: 4.43 g MS salts with vitamins (Cat# M519, Phytotechnology, http://www.phytotechlab.com/), 30 g maltose, 500 mg casein hydrolysate (casamino acids), 1 ml 0.6 mg/ml CuSO4, adjusted to pH 5.8 with 0.1 N KOH, for plates 2.15 g phytagel (Cat# P-8169, Sigma, http://www.sigmaaldrich.com/) was added. After autoclaving, 0.5 ml of 5 mg/ml 2,4-D stock solution was added). Plates were incubated at 28 °C in the dark. The first subculture was 4-5 weeks after the embryos were plated. Subsequent callus transfers, if necessary, were made at 2-3 week intervals (Figure 1b).

Plants were regenerated using the same media used for *B* *. distachyon* (per L: 4.43 g Linsmaier & Skoog (LS) basal medium (Cat# L689, Phytotechnology, http://www.phytotechlab.com/), 30 g maltose, 2 g phytagel, pH 5.8; after autoclaving, 1.0 ml of sterile 0.2 mg/ml kinetin stock solution was added) [34]. Plates were incubated in the light (cool-white fluorescent lighting at a level of 65 μEm^-2^s^-1^ with a 16 hr light: 8 hr dark cycle) at 28 °C. Callus greening and shoot formation occurred in 2-8 weeks. Plantlets were moved to growth media as described previously [32]. Briefly, plantlets were transferred to sundae cups made for food service applications (Cat. # SOL-TS5 and SOL-DL-100, Solo Corporation, http://www.solocup.com/) containing MS sucrose medium (per L: 4.42 g Murashige & Skoog (MS) basal medium with vitamins (Cat # M519, Phytotechnology, http://www.phytotechlab.com/), 30 g sucrose, and 2 g phytagel, pH 5.7) and incubated in the same conditions used for regeneration. When plantlets were approximately 2-5 cm tall, they were transplanted to soil and placed in a growth chamber. Plants were not covered or otherwise gradually introduced to lower humidity conditions.

### Transformation with *Agrobacterium*

Callus was co-cultivated with *Agrobacterium* strain AGL1 [35] containing the construct of interest as previously described [32]. Briefly, *Agrobacterium* was heavily streaked on plates containing MG/L supplemented with the appropriate antibiotics and grown overnight at 28 °C. The bacteria were scraped off the plates using a 1 ml pipette tip and resuspended in liquid callus inducing media containing 200 µM acetosyringone to OD_600_ = 0.6. Then 10 ul of 10% synperonic PE/F68 (Sigma, old name pluronic F68) per 1 ml inoculation media was added (final concentration 0.1%). The callus pieces were then placed in the bacterial suspension and incubated with occasional mixing for 5 minutes. The callus and suspension were then poured into a sterile petri plate and as much suspension as possible was removed with a 1 ml pipette. The callus was then spread on sterile, dry filter in a petri plate (Figure 1c). Plates were closed with parafilm and incubated in the dark at 22 °C for 3 days. The calluses were then placed onto callus inducing media containing 150 mg/L timentin and 40 mg/L hygromycin B. Approximately 25 callus pieces were placed on each 10 cm petri plate. The callus was subcultured onto the same media after 2 weeks of incubation in the dark at 28 °C. After another 2 weeks the callus was placed on regeneration media containing 150 mg/L timentin and 40 mg/L hygromycin B. Shoots were transferred to the MS media described above plus 150 mg/L timentin as soon as they were large enough to handle (Figure 1d).

### Transgene detection and segregation

The transgenic nature of the regenerants was determined using PCR. Leaf samples were tested for the presence of the HptII gene using the primers Hyg1000F (5’ atgaaaaagcctgaactcaccgcgac 3’) and Hyg1000R (5’ ctatttctttgccctcggacgagtgc 3’) and the Phire plant direct PCR kit (Thermo Scientific, http://www.thermoscientific.com) per manufacturer’s instructions.

We used GUS staining to follow the segregation of the transgenes in the T_1_ generation and in the outcrossing experiment. Leaf pieces, about 1 cm, were placed in a GUS staining solution (0.1 M sodium phosphate pH 7.0, 0.5 mM potassium ferrocyanide, 0.5mM potassium ferricyanide, 1.5 g/L of X-Gluc (5-bromo-4-chloro-3-indolyl-β-D-glucuronic acid), 0.5% (v/v) Triton X-100) and incubated overnight at 37 °C [36].

### Mitotic chromosome preparation

Mitotic chromosome spreads were generated following a protocol by Zhang and Friebe [37] with a few modifications as described in [38].

## Results and Discussion

**Figure 2:**
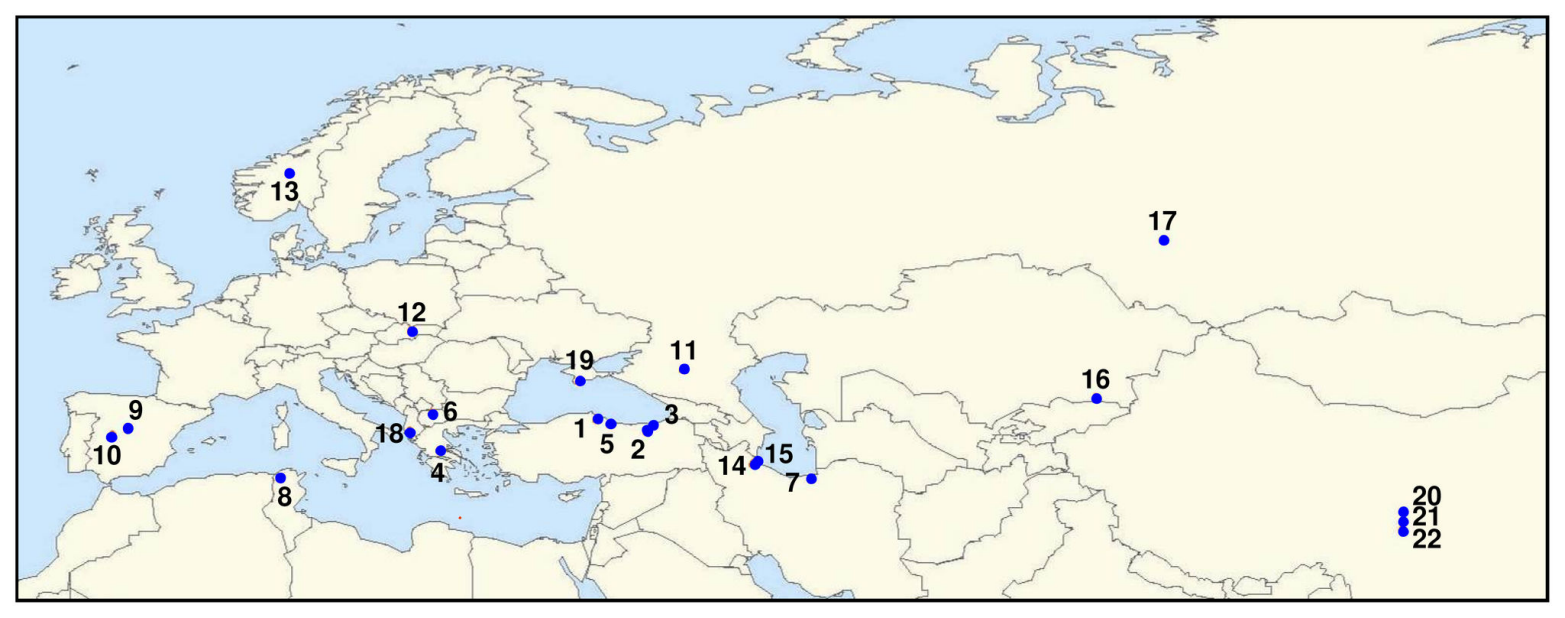
Approximate collection locations for the lines used in this study. All locations were inferred from the collection information found in the Plant Inventory accessed through the GRIN database. For most lines the town nearest the collection site was mapped. Locations 20-22 were mapped using the GPS coordinates of the collection locations. For location 4 and 12 only the country of origin was listed so the map location indicated is the approximate center of the country. See Table 1 for location number to accession assignments. For complete collection data see Figure S1.

**Figure 3:**
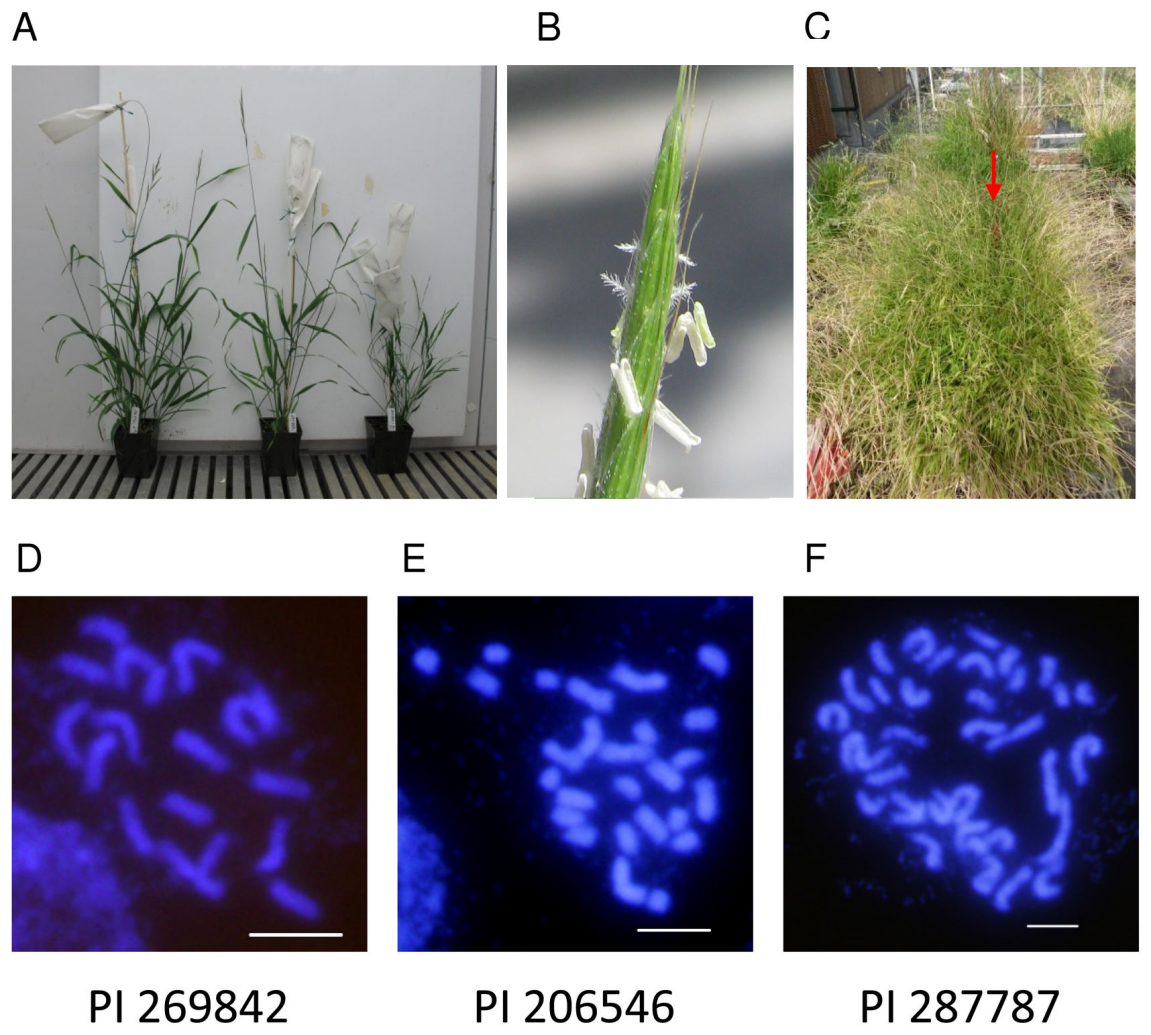
Growth habit and chromosomes. (a) Flowering plants in the growth chamber. The accessions are, from left to right, PI296842, PI297868 and PI204863. Note the variation in height. The pots are 10 cm tall. (b) Close up of flowers. Note the fully exerted anthers and stigmas. (c) Bench full of transgenic plants surrounding a non-transgenic plant to determine pollen flow. The red arrow is pointing to a red stake in the pot of the non-transgenic plant. (d-f) Mitotic chromosome spreads of the indicated accessions. Scale bars = 5 µm.

Twenty-three accessions from a broad geographic region were ordered from the National Plant Germplasm System (Table 1, Figure 2, Figure S1). C-values were determined for 21 accessions and they fell into three groups (Table 1). Seventeen lines had c-values consistent with previous reports, three lines had c-values approximately 1.5 times as large and one line had a c-value approximately twice the size of previous reports [39]. Based on the *B* *. distachyon* controls run at the same time, the approximate genome sizes of the three classes are 340, 510 and 680 Mb. Chromosome counts of representatives of the 1.0, 1.5 and 2.0 c-value groups were 18, 27 and 36, respectively (Table 1, Figure 3d-f). Previous reports indicate most *B* *. sylvaticum* accessions have a chromosome number of 18 [39,40,41]. Higher chromosome numbers, 28, 42, and 56, have also been reported but these were either presented as unpublished data [39] or had ambiguity about the species [40]. Since the vegetative morphology of the larger c-value groups was distinct (not shown) and since they did not flower under the conditions used, we cannot be certain that they are indeed *B* *. sylvaticum* . Thus, we set the four lines with higher chromosome counts and c-values aside and focused on the accessions with a c-value of approximately 1.0.

The plants flowered when approximately 30-50 cm tall but continued to grow and achieved maximum inflorescence heights of 72-132 cm (Table 1). To determine flowering time, seeds were first vernalized by sowing in moist soil and placing the pots in the cold for 2 weeks. The pots were then placed in a growth chamber with 20 hour daylight conditions. Lines that flowered under these conditions did so from 5-10 weeks after transfer to the growth chamber. Ten lines, including all those with higher c-values, failed to flower under these conditions even after 14 weeks (Table 1). We did not attempt to induce flowering with longer vernalization or shifts in day length, as our goal was to identify lines that could be used under our existing conditions. After flowering and maturing seeds, plants largely ceased growth and flowering and began to turn yellow suggesting that they were entering dormancy despite the unchanging environmental conditions. However, at this stage the plants were pot bound so nutritional deficiency may have contributed to the yellowing. Nevertheless, the cessation of growth and flowering suggests that *B* *. sylvaticum* has a strong internal cue to stop growth after setting seed. Placing the plants in 4°C for a few weeks or dividing the plants stimulated new growth and flowering (not shown).

Morphologically, the collection was quite varied in terms of height (Figure 3a, Table 1), stature (upright or prostrate), vestiture (glabrous to pilose) and stem thickness. In addition, five lines (PI172383, PI380758, W6 23501, W6 23524, W6 23538) tended to turn brown and unhealthy. We did not investigate the cause of the browning, but since it showed up repeatedly only in these lines and did not spread to adjacent pots of other lines, it may not be due to a pathogen. The phenotypic variation within this initial collection will serve as a valuable resource to study many traits of interest.

**Figure 4:**
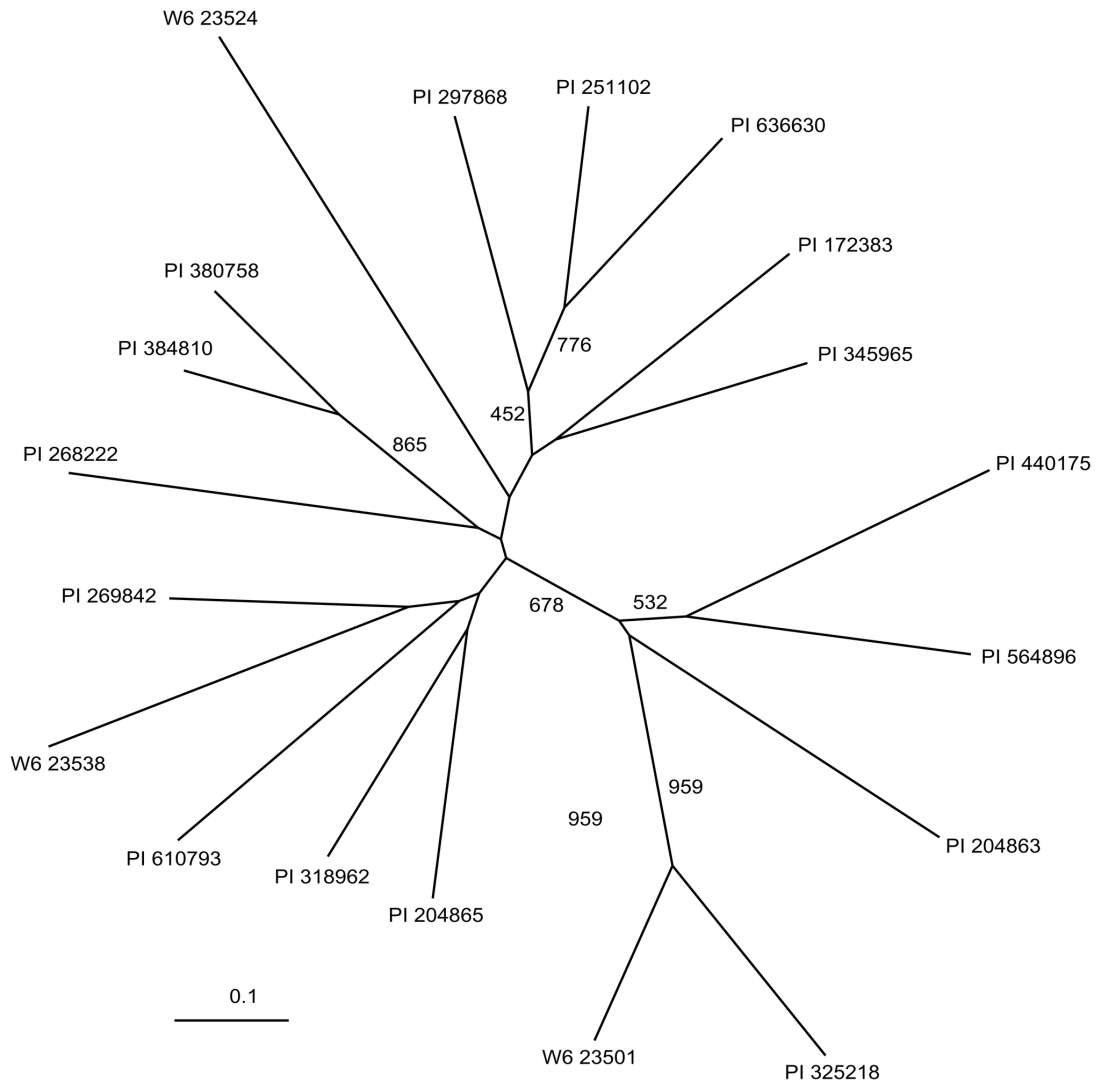
Unrooted neighbor joining consensus tree of 19 lines based on 1,000 shared allele bootstrap trees constructed using 10 SSR markers. Bootstrap values greater than 400 are shown.

To estimate the genetic diversity of the collection, we analyzed the accessions with c-values of ~1 using 10 SSR markers (Table 2, Figure 4). Nine of these markers were developed for *B* *. sylvaticum* [42] and one was developed from *B* *. distachyon* [33]. Interestingly, BDSSR374 was the only *B* *. distachyon* marker that successfully amplified *B* *. sylvaticum* sequence out of 10 markers tried (not shown). However, since the majority of those markers were derived from non-coding sequences, this is not indicative of the similarity of the coding portion of the *B* *. distachyon* and *B* *. sylvaticum* genomes. There was extensive genetic diversity with an average of 11.5 alleles per marker. The average heterozygosity was 19%, indicating that the plants outcross a significant percentage of the time. This is not surprising given that, unlike *B* *. distachyon* , the flowers open and the anthers exert to shed copious pollen (Figure 3b).

**Table 2:** SSR Marker summary.

**Marker**	**Number of Alleles**	**Major Allele Frequency**	**Number of Genotypes**	**Heterozygosity**
**BDSSR374**	4	0.73	4	0
**2-6e6**	12	0.2	15	0.23
**3-2e3**	8	0.36	7	0.05
**6H1**	15	0.14	15	0.23
**6H8**	16	0.23	16	0.14
**2G2**	5	0.43	8	0.18
**4F9**	9	0.52	9	0.45
**6C3**	16	0.14	16	0.18
**2B2**	10	0.43	11	0.14
**3A1**	20	0.14	18	0.32
**Mean**	11.5	0.33	11	0.19

To examine the relationship between accessions, we created an unrooted neighbor joining tree based on 1,000 bootstrap trees (Figure 4). Overall the bootstrap values were low, indicating that there was little correlation between alleles from different markers despite the considerable diversity observed. This may be an artifact due to the small marker set employed, the selection of highly variable SSR markers or may be due to significant interbreeding between the populations sampled. Interestingly, there was little correlation between collection location and genotype. Indeed, three accessions (W6 23501, W6 23524 and W6 23538) collected from nearby locations in China were dispersed throughout the SSR tree (Figure 4). Interestingly, lines collected from distant locations (e.g. W6 23501 and PI325218) were placed nearby on the SSR tree with high bootstrap support. Only two lines (PI380758 and PI384810) collected from nearby locations were placed on the tree in nearby positions with high bootstrap confidence. Similar genetic diversity in small geographic regions and genetic similarity of distantly collected accessions has been observed for *B* *. distachyon* and suggests significant long-distance seed dispersal, presumably mediated by hairs on the seeds [33]. However, since this study examined a small number of markers and a limited germplasm collection, we cannot draw strong conclusions at this time.

We tried to generate embryogenic callus from seven accessions (PI172383, PI204863, PI204865, PI251102, PI269842, PI 29786, PI318962) using immature embryos of various sizes as initial explants and several different callus-inducing media. For initial experiments we used the callus-inducing media used for *B* *. distachyon* [32]. Similar to *B* *. distachyon* , only small immature embryos reliably generated embryogenic callus. During these initial tests we tried adding 500 mg/L casein hydrolysate to the callus inducing media as a source of vitamins and amino acids because it had been shown to improve callus induction in deep water rice varieties [43]. This qualitatively improved the callus so we included casein hydrolysate in all subsequent media tested. We next compared three different combinations of major salts and sugar: LS salts plus sucrose (the standard for *B* *. distachyon* ), MS salts plus sucrose and MS salts plus maltose. In all cases, we qualitatively examined callus quality using a dissecting microscope. PI297868 callus looked the same on MS maltose and LS sucrose. PI204863, PI204865 and PI269842 produced the best embryogenic callus on MS maltose. PI204963 died on LS sucrose. Based on these results, we selected MS salts plus maltose for our transformation experiments.

We next attempted to transform the four accessions (PI204863, PI204865, PI297868 and PI269842) that produced embryogenic callus. Only PI269842 was reproducibly transformed (Figure 1e). However, we did produce a single transgenic plant from PI297868 suggesting that it may be possible to improve the transformation rate for this accession by further method optimization. Despite producing high quality callus that readily regenerated, PI204863 surprisingly does not seem amenable to transformation. All callus for this accession died rapidly on selection, possibly indicating that PI204863 is unusually sensitive to hygromycin. To explore this further, we grew PI204863 on several concentrations of hygromycin side by side with PI269842 and it did not appear that PI204863 was unusually sensitive. Thus, the poor performance of PI204863 must be due to another factor.

Our initial transformation with PI269842 was extremely efficient with an efficiency of 168% (Table 3). Efficiency was calculated as the percentage of callus pieces initially co-cultivated with *Agrobacterium* that later produce transgenic lines. It is possible to get greater than 100% efficiency because the initial calluses break into smaller pieces when placed and shaken in the *Agrobacterium* suspension. Since the callus splitting occurs before transformation, each transgenic line arising from a callus piece after co-cultivation is an independent event. We noticed that *B* *. sylvaticum* callus was more friable than *B* *. distachyon* callus and, on average, the number of callus pieces after co-cultivation was approximately five times the number of callus pieces initially incubated with *Agrobacterium* . However, many of those pieces were very small and difficult to count. We repeated the transformation with PI269842 two additional times and with a PI269842 inbred line, Ain-1, that had been inbred for three generations. Overall transformation efficiency varied from 16 to 168% with an average of 67%. For the last and largest transformation it was necessary to split the calluses into three plates for co-cultivation. Upon removing the plates from 3 days of co-cultivation on filter paper we noticed that the calluses and filter paper in two plates were quite moist and that the calluses and filter paper in the third plate were very dry. This difference may be due to the order in which the calluses were placed onto the filter paper after removal of the *Agrobacterium* suspension. The callus on top of the pile may have been dryer than the callus on the bottom, which was still in contact with some residual *Agrobacterium* suspension. In addition, there was more condensation on top of the plate with the dry callus. The condition of the callus was monitored after co-cultivation and we saw that the dry calluses appeared much healthier and produced approximately seven times as many transgenic plants as the wetter callus pieces. We suspect that much of the variability observed in transformation efficiency is due to the dryness of the calluses and suggest blotting the callus dry on sterile filter paper prior to the 3-day incubation on sterile filter paper in future transformations, as we now do for *B* *. distachyon* (unpublished).

**Table 3:** Transformation of accession PI269842 and the inbred line Ain-1.

Inbred line	Transformation number	Construct	Number of calluses co-cultivated	Number of transgenic plants	Efficiency^1^
not inbred	1	pOL001	50	84	168%^2^
not inbred	2	pJJ2LB	64	10	16%
not inbred	3	pJJ2LB	69	43	62%
Ain-1	4	pJJ2LB	236	51	22%^3^
				average	67%

^1^Efficiency was calculated by dividing the number of transgenic plants by the number of co-cultivated calluses.

^2^Since the calluses broke into smaller pieces immediately when mixed with *Agrobacterium* , more that one independent event can arise from one initial callus and efficiencies greater than 100% are possible. On average, the number of callus pieces after co-cultivation was five times the number of initial callus pieces. Only one transgenic line per callus piece after co-cultivation was counted.

^3^The calluses were split into three portions for co-cultivation. One portion was much drier than the other two after co-cultivation. Since 70% of the transgenic lines were derived from dry calluses and 25% of the calluses after co-cultivation were dry calluses, the dry callus efficiency is approximately 61%.

PCR of young leaf samples from T_0_ plants was used to determine the transgenic status of 96 regenerants. Amplification of the HptII gene confirmed that 99.0% of the regenerant plants were transgenic (data not shown). To estimate the number of insertions in a given transgenic line, we examined the segregation of β-glucuronidase (GUS) expression in the T_1_ generation of nine lines. A chi square test was consistent with a single insertion for five of the nine lines (Table 4). Thus, similar to *B* *. distachyon* , rice and *Arabidopsis* , each transgenic line likely has insertions at 1-2 genetic loci [34,44,45,46].

**Table 4:** Segregation of GUS expression in the T_1_ generation of PI269842 transgenics.

**Transformation event**	**GUS positive plants**	**GUS negative plants**	**χ^2^ value for 3:1 ratio**	**p-value**
4	30	4	3.2	0.07^1^
15	29	9	0.0	0.85^1^
19	29	6	1.2	0.28^1^
23	31	7	0.9	0.35^1^
30	17	16	9.7	0.001^2^
58	45	1	12.8	0.0003^2^
64	38	4	5.4	0.02^2^
67	37	0	12.3	0.0004^2^
70	35	5	3.3	0.07^1^

^1^Consistent with a 3:1 segregation ratio.

^2^Not consistent with a 3:1 segregation ratio.

With a greenhouse full of T_0_ plants, we had the perfect opportunity to study gene flow due to outcrossing under greenhouse conditions. To do this we placed a non-transgenic plant in the middle of a bench of transgenic lines (Figure 3c). We then assayed GUS expression in seedlings resulting from the seed produced to infer the rate of outcrossing (Figure 1e). We observed GUS expression in four out of 148 progeny from the non-transgenic plants (2.7%). Since the T_0_ plants are hemizygous, the actual outcrossing rate is probably at least 2-fold higher or at least 5.4%. While not a firm estimate of outcrossing under ideal or natural conditions, this experiment clearly demonstrates the need to bag inflorescences prior to anthesis to ensure self-set seed. It also highlights a difference with the selfing species *B* *. distachyon* , whose flowers rarely open and for which no outcrossing was observed in a similar experiment [33].

With a highly efficient transformation protocol and inbred lines for *B* *. sylvaticum* in hand, this little perennial grass can now serve as a tractable perennial model system. This fills a pressing need due to the increasing interest in perennial grasses as bioenergy crops and the potential ecological and agricultural benefits promised by the development of perennial grains. When paired with *B* *. distachyon* , we have complementary model systems that can be used to efficiently probe the genetic basis of perenniality. Another promising use for *B* *. sylvaticum* is to study mechanisms of sterility and develop gene containment systems. The perennial nature will give researchers unlimited time to characterize sterile lines (including outcrossing studies) and the larger flowers that open to shed pollen will simplify pollination-related studies.

## Supporting Information

Figure S1 **Collection data compiled from the plant introduction information for each accession.**
(PDF)
