# Supplementary material for: Brachypodium sylvaticum, a Model for Perennial Grasses: Transformation and Inbred Line Development
Source: PLoS One. 2013 Sep 20;8(9):e75180. doi: 10.1371/journal.pone.0075180 (PMC3779173; doi:10.1371/journal.pone.0075180)
Supplement: Figure S1 — Collection data compiled from the plant introduction information for each accession. (PDF) [file pone.0075180.s001.pdf]

Supplemental figure 1. Collection data for accessions used in this study. Excerpts were extracted from the Germplasm Resources Information Network (GRIN) online database http://www.ars-grin.gov/. For most accessions excerpts were taken from the original Plant Inventory lists obtained as a PDF file from GRIN.

172354 to 172491.

From Turkey. Seeds collected by Jack R. Harlan, Agricultural Explorer, Bureau of Plant Industry, Soils, and Agricultural Engineering, Beltsville, Md. Received Jan. 4, 1949.

Seeds collected in 1948.

172383. BRACHYPODIUM SYLVATICUM (Huds.) Beauv. Poaceae.

No. 6695. Congara, Gerze, Sinop. July 24.

173614 to 173831.

From Turkey. Seeds presented by Jack R. Harlan, Agricultural Explorer, Bureau of Plant Industry, Soils, and Agricultural Engineering, Beltsville, Md. Received Feb. 7, 1949.

Collected in 1948.

173700. BRACHYPODIUM SYLVATICUM (Huds.) Beauv. Poaceae.

No. 7854. Closed mountain valley above Hakari and below Asagi Kanonos, Hakari. Aug. 25.

204853 to 204935.

From Turkey. Seeds collected by R. K. Godfrey, Agricultural Explorer, Bureau of Plant Industry, Soils, and Agricultural Engineering, Beltsville, Md. Received Jan. 22, 1953.

Collected in 1952.

204863. BRACHYPODIUM SYLVATICUM (Huds.) Beauv. Poaceae.

No. 685. Zigana Pass between Torul, Gumusane, and Macka, Trabzon. Sept. 21.

204865. BRACHYPODIUM SYLVATICUM (Huds.) Beav. Poaceae.

No. 713. Near sea level, Trabzon. Sept. 23. Perennial.

206544 to 206598.

From Greece. Seeds presented by Marvin Klemme, Range Management Specialist, MSA, and Panos Margaropoulos, Forest Specialist, Greek Ministry of Agriculture, Athens. Received Mar. 31, 1953.

206546. BRACHYPODIUM sp.

No. 1.

206617 to 206780.

From Turkey. Seeds collected by R. K. Godfrey, Agricultural Explorer, Bureau of Plant Industry, Soils, and Agricultural Engineering, Beltsville, Md. Received Apr. 1, 1953.

206619. BRACHYPODIUM SYLVATICUM (Huds.) Beauv. Poaceae.

No. 757. Near Samsun. Sept. 29. Annual.

251095 to 251151.

From Yugoslavia. Seeds collected by H. S. Gentry, agricultural explorer, New Crops Research Branch, Crops Research Division, Beltsville, Md. Received Sept. 12, 1958.

251102. BRACHYPODIUM SYLVATICUM (Huds.) Beauv. Poaceae.

Col. No. 17180. Road bank six miles north of Veles, Macedonia. Elevation 1,000 feet. Graceful grass; unpalatable.

268205 to 268323.

From Iran. Seeds collected by J. R. Harlan, agricultural explorer, New Crops Research Branch, Plant Industry Station, Beltsville, Md. Received Sept. 30, 1960.

268222. BRACHYPODIUM cf. SYLVATICUM (Huds.) Beauv.

Col. No. 143. Mountains 172 km. east of Gorgan. Plants 0.5 meter high.

269842 to 269858.

From Tunisia. Seeds presented by the United States Operations Mission, Tunis. Received Nov. 28, 1960.

269842. BRACHYPODIUM SYLVATICUM (Huds.) Beauv. Poaceae.

Fifteen km. north of Ain Draham; about 75 cm. annual rainfall. Plants slender; heads dropping; foliage light green.

287737 to 287999.

From Spain. Seeds presented by the Instituto Forestal de Investigaciones y Experiencias, Madrid, via the United States Embassy, Rome, Italy. Public Law 480 Project E–25–FS–1. Received Mar. 27, 1963.

287787. BRACHYPODIUM SYLVATICUM (Huds.) Beauv.

G–7. Toledo Prov.

297867 to 298016.

From Australia. Seeds presented by the Commonwealth Scientific and Industrial Research Organization, Canberra. Received May 15, 1964.

297868. AGROPYRON CANINUM (L.) Beauv. Gramineae.
Awned wheatgrass.

C.P.I. 28473.

318922 to 319147.

From Spain. Seeds presented by the Institute Forestal de Investigaciones y Experiencias, Madrid, via the United States Embassy, Rome, Italy. Public Law 480 Project E–25–FS–1. Numbered Mar. 13, 1967.

318962. BRACHYPODIUM SYLVATICUM (Huds.) Beauv.

F–88. Candeleda, Ávila. July 1962.

325178 to 325526.

From the Union of Soviet Socialist Republics. Seeds collected by Willis H. Skrdla, agricultural explorer, Crops Research Division, Regional Plant Introduction Station, Ames, Iowa. Numbered Feb. 2, 1968.

325218. BRACHYPODIUM SYLVATICUM (Huds.) Beauv. **Falsebrome grass.**

Col. No. S–218. Stavropol region. Near northern orphanage on Klukhov River. Elevation 1,556 m. Seed from several plants. Leaves 25 to 35 cm. high; 6 to 7 mm. wide.

344555 to 344592.

From Czechoslovakia. Seed presented by Botanicka Zahrada, University P. J. Safarika, Kosice. Numbers refer to those in 1968 Index Seminum, XVIII, of Botanicka Zahrada. Received August 10, 1969.

344569. BRACHYPODIUM SILVATICUM (Huds.) Beauv. Gramineae.
Falsebrome.

167. Bromus commutatus Schrad.

Note the handwritten text refers to the next entry, not 344569

345948 to 346070.

From Norway. Seed presented by Botanical Garden, University of Oslo, Oslo. Code numbers refer to those in 1969 Index Seminum. Source data given if not of Garden origin. Received September 10, 1969.

345965. BRACHYPODIUM SYLVATICUM (Huds.) Beauv. **Falsebrome.**

1792.

380603 to 381079.

From Iran. Seed collected by J. L. Schwendiman, Plant Materials Center, Soil Conservation Service, Pullman, Washington. Received September 15, 1972.

380758. BRACHYPODIUM SYLVATICUM (Huds.) Beauv. Gramineae.

False bromegrass.

416. Ardebil, on east side of grade to Astara. Perennial pale green leaves,

384730 to 384981.

From Iran. Seed collected June-July 1972 by D. R. Dewey, Crops Research Laboratory, Logan, Utah. Received October 15, 1972.

384810. BRACHYPODIUM sp.

D-1178. Toward Astara, 40 km northeast of Ardebil. Elevation 1,500 m. Plants with slender, drooping culms to 80 cm. Pubescent leaves and spikes. Bulk collection on Caspian slope. Wild.

440174 TO 440178. Brachypodium sp. (Poaceae).

From Soviet Union. Collected by D.R. Dewey and A.P. Plummer,

440174 TO 440178-continued

Crops Research Lab., Utah State Univ. & Intermountain Forest & Range Experiment Station, USDA, Logan and Ephraim, Utah. Received February 1978.

440175. D-1861. Collected August 12, 1977. Gorky Amusement Park, Alma Ata. 800m. Shade. Leaves broad, dark green. Culms to 80cm long. Spikes pendulous. Wild. Seed.

PI 564896
Brachypodium sylvaticum (Huds.) P. Beauv. POACEAECollected in: Russian Federation Maintained by the Western Regional PI Station. NPGS received: 16-Jan-1992. PI assigned: 1993. Inventory volume: 202. Life form: Perennial. Improvement status: Wild material. Form received: Seed. Accession backed up at second site.

Accession names and identifiers
DJ-4194
Idtype: DONOR. Group: USDA-ARS-FRRL. Comment: USDA-ARS Forage Range Research Laboratory Logan Utah.
W6 10257
Idtype: SITE. Group: W6. Comment: W6 accession numbers. Source: USDA, ARS, WRPIS.
Availability
Material is available for distribution. The normal amount distributed is 250 seeds.
Request this germplasm
Narrative
Leaves broad, light-green. Single spikelets. Considered to be a rare and endangered species.

Annotations
ActionDateSiteOld NameRe-identification03-Sep-2007W6Brachypodium sp.Source History
Accession was collected. 28-Aug-1989. Russian Federation.
Locality: From field plots at Central Siberian Botanical Garden, Academy Town, Novosibirsk. Siberia.
Collectors:
Dewey, D., USDA-ARS.
Jensen, K., USDA, ARS.

Accession was donated. 16-Jan-1992. Utah, United States.
Donors:
Jensen, K., USDA, ARS.

Cite as: USDA, ARS, National Genetic Resources Program. *Germplasm Resources Information Network - (GRIN)*. [Online Database] National Germplasm Resources Laboratory, Beltsville, Maryland. Available: http://www.ars-grin.gov/cgi-bin/npgs/acc/display.pl?1459867 (15 November 2011)

PI 610793
Brachypodium sylvaticum (Huds.) P. Beauv. POACEAECollected in: Albania
Maintained by the Western Regional PI Station. NPGS received: Sep-1996. PI assigned: 1999. Inventory volume: 208. Life form: Perennial. Improvement status: Wild material. Form received: Seed. Accession backed up at second site.

Accession names and identifiers
AI 028
Idtype: COLLECTOR.
W6 18620
Idtype: SITE. Group: W6. Comment: W6 accession numbers. Source: USDA, ARS, WRPIS.
Availability
Material is available for distribution. The normal amount distributed is 250 seeds.
Request this germplasm
Narrative
Height 40-60cm.

Annotations
ActionDateSiteOld Name Re-identification11-Jun-2001W6Lolium perenneRe-identification03-Sep-2007W6Brachypodium sp.**Source History**
Accession was collected. Sep-1996. Albania.
Locality: Jonufer, S of Vlore, off the Adriatic Bay of Vlore. Habitat: West facing slope in terraced olive orchard.. Latitude: 40 deg 23 min 56 sec N (40.39888889), Longitude: 19 deg 28 min 38 sec E (19.47722222) (GPS coordinates) GoogleMap it. Elevation: 50 meters
Collectors:
Garvey, E., USDA, ARS.
Muehlbauer, F., USDA, ARS.
Xhuveli, L., Agricultural University of Tirana.
Cite as: USDA, ARS, National Genetic Resources Program. Germplasm Resources Information Network - (GRIN). [Online Database] National Germplasm Resources Laboratory, Beltsville, Maryland. Available: http://www.ars-grin.gov/cgi-bin/npgs/acc/display.pl?1532162 (15 November 2011)

PI 636630
Brachypodium sylvaticum (Huds.) P. Beauv. POACEAECollected in: Krym, Ukraine
Maintained by the Western Regional PI Station. NPGS received: 15-Aug-1999. PI assigned: 2005. Inventory volume: 214. Life form: Perennial. Improvement status: Wild material. Form received: Seed.

Accession names and identifiers
UKR-99-040
Idtype: COLLECTOR. Comment: Collection site 3
W6 21725
Idtype: SITE. Group: W6. Comment: W6 accession numbers. Source: USDA, ARS, WRPIS.
Intellectual Property and Material Transfer Agreements
MOU between the U.S. and the Ukraine
Date issued: 18-Jun-1999. Comment: Agreement number 58-1275-9M-F111
View MTA disclaimer

Availability
Material is available for distribution. The normal amount distributed is 250 seeds.
Request this germplasm
Source History
Accession was collected. 28-Jul-1999. Krym, Ukraine.
Locality: Near Black Sea and Sanatome along road A-294. Habitat: South slope, rocky, dry..
Latitude: 44 deg 24 min 15 sec N (44.40416667), Longitude: 33 deg 49 min 30 sec E (33.825) (GPS coordinates) GoogleMap it. Elevation: 230 meters
Collectors:
Bockelman, H., USDA, ARS.
Boguslavsky, R., National Centre for Plant Genetic Resources of Ukraine.
Johnson, R., USDA, ARS.
Korzhenevsky, V., State Nikitsky Botanical Gardens.

Observations
Vouchers for accession
Computer image.
Taken by: Johnson, R., USDA, ARS. On: 10/15/1999.
collection site. Inventory sample: PI 636630 SD 99o.
Comment: Collection site 3.

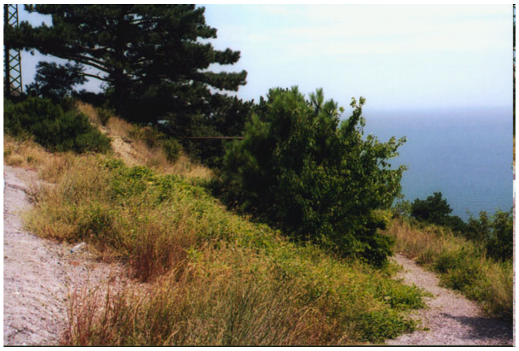

Computer image.

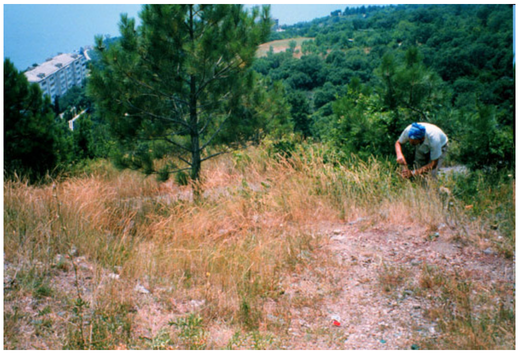

Taken by: Johnson, R., USDA, ARS. On: 10/15/1999.
collection site(b). Inventory sample: PI 636630 SD 99o.
Comment: Collection site 3.

Cite as: USDA, ARS, National Genetic Resources Program. Germplasm Resources Information Network - (GRIN). [Online Database] National Germplasm Resources Laboratory, Beltsville, Maryland.
Available:
http://www.ars-grin.gov/cgi-bin/npgs/acc/display.pl?1584811 (15 November 2011)
